# Osteoporosis, vertebral fractures and metabolic syndrome in postmenopausal women

**DOI:** 10.1186/1472-6823-14-93

**Published:** 2014-12-10

**Authors:** Abdellah El Maghraoui, Asmaa Rezqi, Salwa El Mrahi, Siham Sadni, Imad Ghozlani, Aziza Mounach

**Affiliations:** Rheumatology Department, Military Hospital Mohammed V, PO Box: 1018, Rabat, Morocco

**Keywords:** Metabolic syndrome, Vertebral fracture assessment (VFA), Dual-energy X-ray absorptiometry (DXA), Osteoporosis, Women, Vertebral fractures

## Abstract

**Background:**

The combined effect of the metabolic syndrome (MS) risk factors on bone health has led to controversial results and it is still not clear whether this effect is protective or detrimental. The study aimed to examine the association between MS and bone mineral density (BMD), osteoporosis, and vertebral fractures (VFs) among ambulatory older postmenopausal women.

**Methods:**

270 post-menopausal women with a mean age of 61.0 years ± 7.8 (50 to 90) with no prior known diagnosis of osteoporosis were recruited. BMD and Lateral vertebral fracture assessment (VFA) images were obtained using a GE Healthcare Lunar Prodigy densitometer. VFs were defined using a combination of Genant semiquantitative approach and morphometry.

**Results:**

The MS as defined by the NCEP-ATP III was present in 62 women (23.0%). According to the WHO classification, 82 had osteoporosis at any site (30.4%). VFs were identified in 116 (43.0%): 80 (29.6%) had grade 1 and 36 (13.3%) had grade 2 or 3. Women with MS had a significantly higher BMD and lower prevalence of osteoporosis (17.7% vs. 34.1%) than those without MS. No significant statistical difference was noted in prevalence of VFs (14.5 vs. 13.0%). There were significantly less women with MS among the group of osteoporotic women (13% vs. 27%; p = 0.018). Conditional regression binary analysis assessing the presence of osteoporosis as the dependent variable showed that women with a MS had a significant 71% decrease in the odds of being osteoporotic by BMD compared with women who had not MS accounting for age, BMI, number of parities and years since menopause.

**Conclusion:**

Women with MS had higher BMD at the hip and spine, suggesting a protective effect of MS on bone. However, the prevalence of VFs was similar between women with or without MS.

## Background

Metabolic syndrome (MS) is a clinical condition composed of anthropometric, physiologic, and biochemical abnormalities predisposing affected individuals to the development of type 2 diabetes and cardiovascular disease. Rather than total adiposity, the core clinical component of the syndrome is visceral and/or ectopic fat (i.e., fat in organs not designed for fat storage), whereas the principal metabolic abnormality is insulin resistance [1]. The major characteristics of this syndrome include abdominal obesity, lipid abnormalities (high serum triglyceride and/or low HDL cholesterol), hypertension, and hyperglycemia. In 2001, the National Cholesterol Education Program-Adult Treatment Panel III (NCEP-ATP III) [2] definition required the presence of at least 3 of the components mentioned above.

The association between each of these risk factors and osteoporosis, a major cause of morbidity and mortality in old age, has been extensively studied, with conflicting results [3–10]. While some studies indicate a possible protective effect of obesity or diabetes on fracture risk, increased fracture risk among diabetics, patients with high blood pressure or patients with high levels of cholesterol and/or triglycerides has been reported in some but not all studies. The combined effect of the MS risk factors on bone health has also led to controversial results. Therefore, it is still not clear whether this effect is protective or detrimental. No clear pattern of bone fractures in MS stands out from the studies undertaken up to now. The concept that MS patients may suffer from fewer fractures than controls seems logical since BMD is often higher. Epidemiological studies, however, are not conclusive. Most do not show differences in the incidence of fractures between patients and controls, but results in either direction have also been reported [11, 12].

Based on these conflicting results, and the high prevalence of osteoporosis, VFs and MS in the aging Moroccan population [13, 14], we conducted a cross-sectional study to examine the association between MS and BMD, osteoporosis, and VFs among ambulatory older postmenopausal women.

## Methods

### Subjects

A total of 270 caucasian postmenopausal women (age range: 50–90 yr) living in the Rabat area participated in the present study. Women were recruited through advertisements and “word of mouth” from june 2012 to march 2013. Original inclusion criteria were age > 50, menopause > 1 year and no previous osteoporotic fracture or known diagnosis of osteoporosis. Women with liver or renal disease, endocrine or metabolic abnormalities, and receiving medicine known to influence bone mineralization, such as corticosteroids, heparin, anticonvulsants, vitamin D, bisphosphonates, were excluded. Our institutional review board (Comité d’éthique et de recherche de l’hôpital Militaire Mohammed V) approved this study. The procedures of the study were in accordance with the Declaration of Helsinki, and local ethics committee approval was obtained for the study (Comité d’éthique de la Faculté de Médecine et de Pharmacie de Rabat). All the participants gave an informed and written consent. Each subject completed a standardized questionnaire designed to document putative risk factors of osteoporosis. The questionnaire collected information on current medication use, history of peripheral traumatic fractures, smoking habits, and level of physical activity in leisure time, along with calcium consumption and the use of vitamins and medications. Height and weight were measured in light indoor clothes without shoes. Body mass index (BMI)] was calculated by dividing weight in kilograms by height in meters squared. Waist was measured in centimeters at the bending point using a flexible tape measure, with the participant wearing single-thickness clothing and standing in an erect position with feet together. Although this is not a population-based cohort, care was taken to ensure representativeness of the general population with a particular regard to the inclusion of a wide range of age categories, body sizes and activities. We did not exclude individuals using inhalation steroids or with certain lifestyle habits such as heavy smoking, being sedentary, being athletic, or having a high or low calcium intake, which are examples of voluntary factors that may have some impact on bone metabolism.

The women were asked whether they usually drank milk or alcohol; if they ate cheese or yogurt; if they did gymnastics or jogging/walking and if they smoked tobacco. If the answer was positive, they were asked to quantify their average current consumption (evaluated on the 7 day prior to the interview) of milk or yogurt (mL/day), cheese (g/day) and wine and/or spirits (mL/day). Tobacco smoking was quantified as average number of cigarettes (smoked/day) multiplied by the number of years of smoking, gymnastics as min/week, or jogging/walking as min/day. Finally, patients were categorized as never smokers, ex-smokers and current smokers; high, normal and low calcium intake (more than 1500 mg/day, between 800 and 1500 day − 1 and below 800 mg/day, respectively); high, moderate and low physical activity (more than 3, 2–3 and below 1 hour/week, respectively).

### Bone mineral density (BMD) measurement

BMD was determined by a Lunar Prodigy Vision DXA system (Lunar Corp., Madison, WI). The DXA scans were obtained by standard procedures supplied by the manufacturer for scanning and analysis. All BMD measurements were carried out by 2 experienced technicians. Daily quality control was carried out by measurement of a Lunar phantom. At the time of the study, phantom measurements showed stable results. The phantom precision expressed as the coefficient of variation was 0.08%. Moreover, reproducibility has been assessed in clinical practice and showed a smallest detectable difference of 0.04 g/cm^2^(spine) and 0.02 (hips) [15, 16]. Patient BMD was measured at the lumbar spine (anteroposterior projection at L1-L4) and at the femurs (i.e., femoral neck, trochanter, and total hip). Using the Moroccan female normative data [17], the World Health Organization (WHO) classification system was applied, defining osteoporosis as T-score ≤ −2.5 and osteopenia as −2.5 < T-score < −1. Study participants were categorized by the lowest T-score of the L1–4 lumbar spine, femur neck, or total femur.

### Vertebral fracture assessment

VFs was classified using a combination of Genant [18] semiquantitative (SQ) approach and morphometry in the following manner: each VFA image was inspected visually by two trained clinicians (IG and AM) to decide whether it contained a fracture in any of the visualized vertebrae and assigned by consensus a grade based on Genant SQ scale, where grade 1 (mild) fracture is a reduction in vertebral height of 20-25%, grade 2 (moderate) a reduction of 26-40%, and grade 3 (severe) a reduction of over 40%. In case of doubt regarding fracture grade, the vertebrae in question was measured using built-in morphometry. Automatic vertebral recognition by the software was used. Positioning of the six morphometry points was modified by one of the two clinicians only when the software failed to correctly recognize vertebral heights. The intra-rater reproducibility of this method was evaluated using the kappa score to 0.90 (p < 0.0001). Subjects with no fractures were included in the non-fracture group, whereas those with grade 1 or higher fractures were included in the fracture group. However, as many studies rarely report mild deformities as “fractures”, and to realize comparisons with the literature, we distinguished the group of women with grade 1 fractures from the grade 2/3 fracture group. The spinal deformity index (SDI), as described by Kerkeni et al. [19], was then calculated by summing in each patient the grade of each vertebra from T4 to L4. In theory, the SDI value can vary between 0 (no fracture) and 39 (all the assessed vertebrae are grade 3).

### Metabolic syndrome

The prevalence of MS and its components were defined by NCEP-ATP III criteria [2]. Participants were classified as having the MS if any three of the following were present: abdominal obesity (waist circumference greater than 88 cm), triglycerides of 150 mg/dL (1.7 mmol/L) or greater, HDL cholesterol levels less than 50 mg/dL (1.29 mmol/L), fasting glucose of 110 mg/dL (6.1 mmol/L) or greater, or blood pressure of 130/85 mmHg or greater. Participants with documented use of antihypertensive medication were categorized as meeting the blood pressure criteria. Diabetes was defined by the American Diabetes Association 1998 guidelines [20] (fasting plasma glucose equal or greater than 126 mg/dL or 2-h plasma glucose in 75 g oral glucose tolerance test equal or greater than 200 mg/dL).

### Statistical analysis

Results are presented as means (SD) for continuous variables and as frequencies for categorical variables. To compare patients with and without MS and patients with or without osteoporosis, chi-square test and Student t-test were used. To compare patients with and without VFs, chi-square test and analysis of variance ANOVA were used.Potential risk factors for osteoporosis were finally entered to a stepwise conditional binary logistic regression analysis and the resulted odds ratios with 95% confidence intervals were reported. The level for significance was taken as p ≤ 0.05. Excel 2010 and SPSS 20.0 were used for statistical analysis.

## Results

### Participants

In this series of 270 women, the mean ± SD (range) age, weight and BMI were 61.0 ± 7.8 (50 to 90) years, 75.5 ± 11.7 (45 to 106) Kg and 32.3 ± 6.5 (19.1 to 54.8) kg/m^2^, respectively (Table 1). Only 4 women (0.4%) were current smokers. Sixty nine (25.6%) women reported a history of traumatic peripheral fracture before the age of 50. Diabetes was present in 79 women (29.3%), hypertension in 94 (34.8%) and dyslipidemia (hypercholesterolemia and/or hypertriglyceridemia) in 66 (24.4%). The MS as defined by the NCEP-ATP IIIwas present in 62 women (23.0%).

**Table 1:** **Descriptive characteristics of the study population (n = 270)**

		Range
Age (yrs): mean (SD)	61.0 (7.8)	50-90
Weight (kg): mean (SD)	75.5 (11.7)	45 – 106
Height (m): mean (SD)	1.55 (0.05)	1.35-1.79
BMI (kg/m^2^): mean (SD)	32.3 (6.5)	19-54
Years of menopause: mean (SD)	11.3 (8.1)	1-40
Number of pregnancies: mean (SD)	5.2 (2.4)	0-13
History of peripheral fractures: n (%)	69 (25.6)	
Low physical activity: n (%)	179 (66.3)	
Low calcium intake: n (%)	127 (47.0)	
Diabetes: n (%)	79 (29.3)	
Hypertension: n (%)	94 (34.8)	
Dyslipidemia: n (%)	66 (24.4)	
Waist circumference (cm): mean (SD)	103 .8 (10.7)	64-134
Hip circumference (cm): mean (SD)	113.1 (12.9)	58-232
Total body fat mass (%): mean (SD)	47.4 (4.9)	31.0 -56.2
Metabolic syndrome: n (%)	62 (23.0)	
Lumbar spine BMD: mean (SD)	0.98 (0.17)	0.62 -1.94
Femoral neck BMD: mean (SD)	0.85 (0.14)	0.09 -1.8
Total hip BMD: mean (SD)	0.92 (0.12)	0.59 - 1.3
Lumbar spine T-SCORE: mean (SD)	−1.45 (1.3)	−4.5 - 2.8
Femoral neck T-SCORE: mean (SD)	−1.42 (1.01)	−3.7 - 1.2
Total hip T-SCORE: mean (SD)	−0.85 (1.04)	−3.6 - 2.3
Osteoporosis (any site): n (%)	82 (30.4)	

### Bone mineral density

The mean BMD was 0.98 (0.17) g/cm^2^ at the lumbar spine, 0.85 (0.14) g/cm^2^ at the femoral neck and 0.92 (0.12) g/cm^2^ at the total hip. The mean T-scores were −1.45 (1.3), −1.42 (1.01) and −0.85 (1.04) respectively. According to the WHO classification, 82women had osteoporosis at any site (30.4%).

### Vertebral visualization and fracture identification on VFA

**Figure 1:**
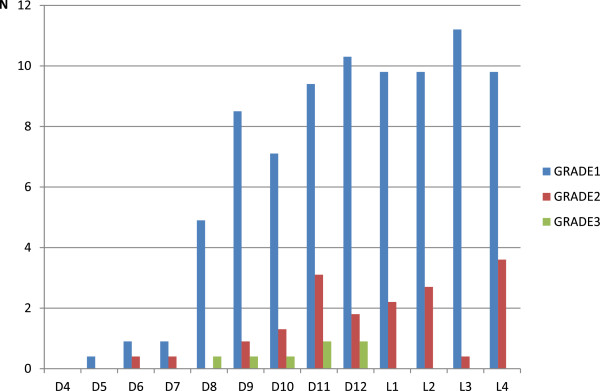
**Vertebral fractures distribution in our study population.**

In these 270 women, 85.7% of vertebrae from T4–L4 and 96.2% from T8–L4 were adequately visualized on VFA. The percentage of vertebrae not visualized at T4, T5, and T6 levels was 51.9%, 40.7, and 26.7% respectively. VFs were identified in 116 (43.0%): 80 (29.6%) had grade 1 and 36 (13.3%) had grade 2 or 3. Among the latter group, 52 (19.2%) women had multiples VFs. Fractures were most common in the mid-thoracic spine and at the thoracolumbar junction (Figure 1). The mean SDI was 1.20 ± 2.2 (0–12).

### Data analysis

Comparison between patients with and without MS (Table 2) showed that those with MS were older, had more pregnancies and longer period since menopause. They had a significantly higher BMD (lumbar spine, femoral neck and total hip) and lower prevalence of osteoporosis as the defined by the WHO criteria (17.7% vs. 34.1%; p = 0.029). No significant statistical difference was noted in prevalence of VFs (14.5 vs. 13.0%; p = 0.672).

**Table 2:** **Comparison between patients with and without metabolic syndrome**

	Metabolic syndrome (N = 62)	Absence of metabolic syndrome (N = 208)	p
Age (yrs): mean (SD)	63.3 (8.4)	60.1 (7.5)	0.013
Weight (Kg): mean (SD)	79.7 (10.2)	74.2 (11.7)	0.001
Height (m): mean (SD)	1.55 (0.05)	1.55 (0.06)	NS
BMI (Kg/m2): mean (SD)	33.2 (6.0)	32.0 (6.6)	NS
Diabetes: n (%)	49 (79.0)	30 (14.4)	0.0001
Hypertension: n (%)	56 (90.3)	38 (18.3)	0.0001
Dyslipidemia: n (%)	40 (64.5)	26 (12.5)	0.0001
Low physical activity: mean (SD)	39 (62.9)	140 (67.3)	NS
Low calcium intake: mean (SD)	30 (48.4)	97 (46.6)	NS
Number of pregnancies: n (%)	6.0 (2.7)	5.0 (2.3)	0.008
Years since menopause (yrs): mean (SD)	12.6 (8.5)	11.1 (7.9)	0.002
Waist circumference (cm): mean (SD)	107.1 (9.7)	102.7 (10.8)	0.002
Hip circumference (cm): mean (SD)	117.1 (17.7)	111.7 (11.8)	0.03
History of traumatic fractures: n (%)	20 (32.3)	49 (23.6)	NS
Lumbar spine (g/cm^2^): mean (SD)	1.025 (0.15)	0.977 (0.17)	0.038
Lumbar spine T-score: mean (SD)	−1.08 (1.2)	−1.57 (1.3)	0.001
Total hip BMD (g/cm^2^): mean (SD)	0.972 (0.13)	0.908 (0.12)	0.003
Total hip T-score: mean (SD)	−0.4 (1.0)	−0.9 (1.0)	0.001
Femoral neck BMD (g/cm^2^): mean (SD)	0.887 (0.18)	0.843 (0.12)	0.094
Femoral neck T-score: mean (SD)	−1.2 (1.0)	−1.4 (1.0)	0.087
Osteoporosis (T-score below −2.5 any site): n (%)	11 (17.7)	71 (34.1)	0.029
Vertebral fractures grade 2–3: n (%)	9 (14.5)	27 (13.0)	NS
Spinal deformity index (SDI): mean (SD)	0.95 (1.6)	1.29 (2.4)	NS

Comparison between patients with and without osteoporosis (T-score below −2.5 in any of the lumbar spine, femoral neck or total hip) showed that those with osteoporosis were older, had lesser weight, height, BMI, and total body fat percentage and had longer period since menopause (Table 3). There were significantly less women with MS among the group of osteoporotic women (13% vs. 27%; p = 0.018).

**Table 3:** **Comparison between patients with and without osteoporosis**

	Osteoporosis (N = 82)	Absence of osteoporosis (N = 188)	p
Age (yrs): mean (SD)	64.0 (8.4)	59.6 (7.5)	<0.0001
Weight (Kg): mean (SD)	70.8 (13.2)	77.5 (10.7)	<0.0001
Height (m): mean (SD)	1.53 (0.05)	1.56 (0.06)	<0.0001
BMI (Kg/m2): mean (SD)	31.0 (6.3)	33.1 (6.7)	0.017
Obesity (BMI > 30 Kg/m2): n (%)	42 (51.2)	107 (56.9)	NS
Diabetes: n (%)	18 (22.0)	61 (32.4)	NS
Hypertension: n (%)	24 (29.3)	70 (37.2)	NS
Dyslipidemia: n (%)	11 (13.4)	55 (29.3)	0.003
Metabolic syndrome: n (%)	11 (13.4)	51 (27.1)	0.018
Low physical activity: mean (SD)	60 (73.2)	119 (63.3)	NS
Low calcium intake: mean (SD)	38 (46.3)	89 (47.3)	NS
Number of pregnancies: n (%)	5.8 (2.7)	5.0 (2.3)	0.016
Years since menopause (yrs): mean (SD)	15.0 (8.9)	9.9 (7.2)	<0.0001
Waist circumference (cm): mean (SD)	103.9 (9.7)	103.6 (10.8)	NS
Hip circumference (cm): mean (SD)	111.1 (17.7)	113.7 (11.8)	NS
Percentage of body fat: mean (SD)	46.1 (6.1)	47.9 (4.0)	0.016
History of traumatic fractures: n (%)	20 (24.4)	49 (26.1)	NS

Comparison between patients without VFs and patients with VFs grade 1 and grade 2/3 (Table 4) showed that women with VFs were older, have a longer period since menopause, were more likely to have history of traumatic peripheral fracture and had a lower BMD at the lumbar spine, femoral neck and total hip. Prevalence of MS was similar between the three groups.

**Table 4:** **Comparison between patients with and without vertebral fractures**

	Absence of VFs (N = 154)	VFs grade 1 (N = 80)	VFs grade 2–3 (N = 36)	p
Age (yrs): mean (SD)	59.6 (7.36)	62.1 (8.4)	64.3 (8.07)	<0.001
Weight (Kg): mean (SD)	75.9 (11.0)	74.8 (12.7)	75.3 (11.7)	NS
Height (m): mean (SD)	1.55 (0.05)	1.56 (0.05)	1.54 (0.06)	NS
BMI (Kg/m^2^): mean (SD)	32.4 (6.5)	32.0 (6.6)	32.6 (6.3)	NS
History of traumatic fractures: n (%)	33 (21.4)	22 (27.5)	14 (38.9)	0.03
Low physical activity: n (%)	102 (66.2)	51 (63.8)	26 (72.2)	NS
Low calcium intake: n (%)	73 (47.4)	36 (45.0)	18 (50.0)	NS
Years since menopause (yrs): mean (SD)	10.1 (7.6)	13.1 (8.4)	13.9 (8.4)	0.003
Number of pregnancies: n (%)	5.0 (2.3)	5.7 (2.4)	5.5 (3.0)	NS
Diabetes: n (%)	54 (35.1)	16 (20.0)	9 (25.0)	NS
Hypertension: n (%)	51 (33.1)	26 (32.5)	17 (47.2)	NS
Dyslipidemia: n (%)	36 (23.4)	22 (27.5)	7 (19.4)	NS
Metabolic syndrome: n (%)	34 (22.1)	19 (23.8)	9 (25.0)	NS
Waist circumference (cm): mean (SD)	103.7 (10.4)	103.9 (10.6)	103.6 (12.3)	NS
Hip circumference (cm): mean (SD)	113.6 (14.3)	111.9 (10.4)	113.0 (10.7)	NS
Lumbar spine BMD (g/cm^2^): mean (SD)	1.011 (0.16)	0.966 (0.19)	0.936 (0.12)	0.029
Lumbar spine T-score: mean (SD)	−1.30 (1.1)	−1.56 (1.6)	−1.83 (1.0)	NS
Total hip BMD (g/cm^2^): mean (SD)	0.943 (0.11)	0.905 (0.15)	0.881 (0.12)	0.013
Total hip T-score: mean (SD)	−0.69 (0.9)	−0.99 (1.2)	−1.22 (1.0)	0.012
Femoral neck BMD (g/cm^2^): mean (SD)	0.869 (0.13)	0.831 (0.13)	0.834 (0.19)	NS
Femoral neck T-score: mean (SD)	−1.25 (0.9)	−1.53 (1.1)	−1.83 (0.8)	0.005
Osteoporosis (T-score below −2.5 any site): n (%)	44 (24.0)	32 (40.0)	14 (38.9)	0.006

*BMI* body mass index; *BMD* bone mineral density; *VFs* vertebral fractures.

Conditional regression binary analysis assessing the presence of osteoporosis as the dependent variable showed that women with a MS had a significant 71% decrease in the odds of being osteoporotic by BMD compared with women who had not MS accounting for age, BMI, number of parities and years since menopause. The predictive variables significantly associated to the osteoporotic status were the presence of MS, BMI, and number of parities (Table 5).

**Table 5:** **Adjusted odds ratios from logistic regression with osteoporosis as the dependant variable**

	OR	IC 95%	p
Metabolic syndrome	0.291	0.130 – 0.651	0.003
Age	1.032	0.977 – 1.091	0.262
Body mass index (BMI)	0.941	0.896 – 0.987	0.013
Years since menopause	1.056	1.000 – 1.115	0.05
Number of pregnancies	1.145	1.008 – 1.290	0.032
Percentage of body fat	0.971	0.910 – 1.037	0.385

Odds ratios are adjusted for age, BMI, years since menopause and number of pregnancies.

## Discussion

In this series of postmenopausal women over 50, MS was significantly and independently associated to higher BMD and lower prevalence of osteoporosis. However, prevalence of VFA-detected asymptomatic VFs was identical in participants with or without MS.

In the aging population, osteoporosis is an important public health problem worldwide due to its high morbidity and mortality [21]. Also, MS is another very common medical problem of epidemic importance, and the number of patients with MS is rapidly increasing in industrialized countries where Western life-style is prevalent.

The prevalence of MS in our study population (23%) was similar to the reported prevalence in adult Caucasians in the United States (20 to 25%) [22], and in Russia [23] (23.1%). Studies of general population demonstrated that the overall prevalence of MS in European countries in women varies from 2.1% in France [24], 14.2% in Finland [25] to 20.9% in Poland [26]. Moreover, it has been shown that the prevalence of MS increases with age and can reach up to 64.4% for women in the 80–89 years of age group [27].

The pathogenesis of MS is multifactorial and progressive. The risk factors of MS are of metabolic origin and consist of abdominal adipose tissue accumulation, atherogenic dyslipidemia, elevated plasma glucose, elevated blood pressure, and a prothrombotic and proinflammatory state. The major risk factors are abdominal obesity and insulin resistance accompanied by increased risk for CVD and type 2 diabetes. Furthermore, aging, physical inactivity, endocrine, and genetic factors exacerbate the MS [28].

Accumulating evidence suggests that individual components of MS such as hypertension, increased triglycerides, and reduced high-density lipoprotein cholesterol are also risk factors for low BMD while other components such as obesity are associated to high BMD.

Many recent publications studied the relation of MS and osteoporosis [29, 30]. In the Rancho Bernardo Study [31], after adjusting for BMI, MS was related to lower BMD. Furthermore, the incidence of osteoporotic non-vertebral fractures was elevated in subjects with MS. In a US population-based study, subjects with MS had an increased femoral neck BMD compared with controls without the syndrome [32]. In the same study, initially unadjusted femoral neck BMD was reduced among persons with MS, but after adjustment for age, gender, and other covariates, it was higher in subjects with MS than in control groups. Moreover, in multivariate linear regression models for each component of MS, femoral neck BMD was significantly higher in subgroups of people with abdominal obesity and diabetes. Hwang et al. [33] reported in 2475 Korean women (21% with MS) that, after adjustment for all covariates, mean vertebral BMD was significantly lower in women with MS. Ahmed and colleagues [34] examined the effect of some components of the MS (BMI, HDL, triglycerides and hypertension) and risk of non-vertebral fractures in 12,780 men and 14,211 women aged 25–98 years (mean = 47 years for both sexes) from the Tromso study, and concluded that increasing burden of MS components protects against non-vertebral fractures. However, participants in this study were young (mean = 47 years) and fractures were not limited to low trauma osteoporotic fractures. Traumatic fractures are more likely to occur in younger participants, and may have a different pathophysiology than osteoporotic fractures. In a recent cross-sectional publication of 2265 women aged over 20 (with prevalence of the MS 5.5% in the premenopausal women and 13.5% in the postmenopausal), Jeon et al. [35] found that women with MS had a lower BMD at the lumbar spine and femoral neck. They also found that the predictive variables for femoral neck BMD were CRP and diastolic blood pressure.

Several factors may explain the controversial results making it difficult to assess whether or not MS and osteoporosis are associated. Although the vast majority of studies addressing bone metabolism in patients with MS have adopted the NCEP-ATPIII criteria, the studied populations are different. Moreover, a certain degree of heterogeneity in their profile is inherent to these criteria. In fact, the diagnosis of MS is made when just three out of five NCEP-ATPIII criteria are satisfied. Consequently, even this definition entails a degree of heterogeneity, as any three or more criteria may be met by each patient. An additional problem is that the confounders used in these studies’ adjustments do not always coincide. This comment is particularly relevant regarding BMI and its related variables (waist perimeter, body weight). Since adjusting for body weight or BMI is, in general, a common procedure in epidemiological studies, results of MS studies are also often adjusted for them. However, such an adjustment distorts the clinical profile of MS, which by definition includes high body weight, waist perimeter or BMI. Hernandez et al. claimed that when adjusting for these, the clinical sense of MS just disappears, or is at least essentially modified [11].

It has been suggested that higher BMD with MS is largely determined by abdominal obesity, and protective effects of fat mass may promote bone formation via high mechanical loading [36], high circulating insulin levels, and factors that are co-secreted with insulin [32]. It is well demonstrated that osteoporosis is linked to inflammation. Previous studies suggest that subjects with high insulin resistance show more inflammation than subjects with low insulin resistance state [37–39] and some studies suggest that this low state of chronic inflammation may impact bone health [40, 41]. Thus the observed conflicting results may be due to the counteracting of a negative effect of low-grade inflammation associated with MS and the protective effect of adiposity [5]. This apparent paradox may be explained by the fact that, at a given level of BMD, MS patients present lower bone quality by means of various mechanisms, including hyperinsulinemia, deposition of advanced glycosylation endproducts (AGEs) in collagen, reduced serum levels of IGF-1, hypercalciuria, renal failure, microangiopathy and inflammation.

Our study has strengths and limitations. The assessment of BMD and fractures was carefully conducted using standard procedures of acquisition, and standard reading of all VFA scans. All the morphometric assessments were made by two experienced investigators after training sessions and after a previous global visualization. The prevalence of obesity and MS was high in our study (64% and 23% respectively) but reflects what is observed in the aging female Moroccan population.The main limitation lies in the procedures used to select subjects, who were all volunteers and ambulatory. The Rabat population may not be adequately representative of the whole Moroccan population. However, since the population living in the area of Rabat is a balanced mixture of the various regions constitutive of the country, we believe the impact on prevalence of VFs or MS estimate is limited. Another limitation is the lack of information about some biological variables that may influence BMD and/or VFs prevalence such as serum 25(OH) vitamin D3 or CRP levels.

## Conclusion

Our study showed that even women with the MS had higher BMD at the hip and spine, suggesting a protective effect of MS on bone, the prevalence of VFs was similar among women with or without MS.
